# Exploring the Association Between Dietary Fruit Intake and Endometriosis: A Systematic Review and Meta-Analysis

**DOI:** 10.3390/jcm14041246

**Published:** 2025-02-13

**Authors:** Pegah Rashidian, Ehsan Amini-Salehi, Shaghayegh Karami, Camran Nezhat, Farr Nezhat

**Affiliations:** 1 Vali-e-Asr Reproductive Health Research Center, Family Research Institute, Tehran University of Medical Sciences, Tehran 1417466191, Iran; pegah.rashidian@rocketmail.com; 2 School of Medicine, Guilan University of Medical Science, Rasht 4144666949, Iran; ehsanaminisalehi1998@gmail.com; 3 Students’ Scientific Research Center (SSRC), Tehran University of Medical Sciences, Tehran 1417466191, Iran; shaghayeghkrm99@gmail.com; 4 Stanford University Medical Center, Palo Alto, CA 94305, USA; camran@camrannezhatinstitute.com; 5 University of California San Francisco, San Francisco, CA 94143, USA; 6 Camran Nezhat Institute, Center for Special Minimally Invasive and Robotic Surgery, Woodside, CA 94061, USA; 7 Nezhat Surgery for Gynecology/Oncology, New York City, NY 10128, USA; 8 Weill Cornell Medical College of Cornell University, New York City, NY 10065, USA; 9 NYU Grossman Long Island School of Medicine, Mineola, NY 11501, USA; 10 Minimally Invasive Gynecologic Surgery and Robotics, NYU Langone Hospital, Long Island, NY 11501, USA

**Keywords:** endometriosis, fruit, diet, dysmenorrhea, antioxidant

## Abstract

**Background/Objectives:** Endometriosis is a chronic gynecological disorder affecting up to 10% of women of reproductive age. The etiology of endometriosis remains unclear; however, there is growing interest in identifying modifiable risk factors, particularly dietary influences. The present study aims to systematically evaluate the association between fruit consumption and the incidence of endometriosis. **Methods:** A comprehensive systematic search was conducted across PubMed, Embase, Web of Science, and Google Scholar for studies published from 1 January 1990 to 30 September 2024. Relevant data were extracted and categorized, and the methodological quality of the included studies was assessed using the Joanna Briggs Institute (JBI) checklists. Additionally, meta-analyses were performed using STATA 18.0 to compare daily and weekly fruit consumption among women with and without endometriosis. **Results:** The analysis included six studies comprising 3689 women with endometriosis and 1463 controls. The meta-analysis revealed no significant association between daily fruit consumption and the risk of endometriosis (odds ratio (OR): 0.95; 95% confidence interval (CI): 0.90–1.01). Similarly, weekly fruit consumption did not demonstrate a significant link to endometriosis risk (OR 1.03, 95% CI: 0.78–1.35). The assessment of publication bias using Begg’s and Egger’s tests, along with contour-enhanced funnel plots, indicated the absence of publication bias in the data across both analysis groups. **Conclusions:** This study indicates that fruit consumption does not significantly influence the risk of developing endometriosis. Additional research is necessary to examine preferred dietary interventions for populations affected by this condition.

## 1. Introduction

Endometriosis is a chronic, inflammatory gynecologic disorder characterized by the presence of endometrial glands and stroma-like lesions outside the uterus, affecting up to 10% of women of reproductive age [1,2]. Of the affected women, approximately 70% experience symptoms such as chronic pelvic pain, dysmenorrhea, dyspareunia, and pain during urination and bowel movements [2,3,4]. These symptoms can significantly impair the quality of life and are often associated with infertility. In severe stages, endometriosis presents challenges similar to other reproductive health conditions that may require additional interventions, such as fertility preservation [5,6,7,8]. Although the precise pathophysiology of endometriosis is still not fully understood, Sampson’s theory of retrograde menstruation is widely accepted as a plausible explanation [9]. Additionally, researchers propose that specific immunological and inflammatory pathways involved in endometriosis may overlap with those in other conditions [10,11]. Due to its substantial impact on women’s health, there is growing interest in identifying modifiable risk factors that could influence the onset and progression of endometriosis.

Dietary factors, particularly antioxidant-rich foods, are increasingly being investigated for their potential impact on endometriosis. Antioxidants, commonly found in fruits and vegetables, may counteract oxidative stress—a key contributor to inflammation in endometriosis [12,13]. Studies such as that of Mier-Cabrera et al. have observed that women with endometriosis who followed diets rich in antioxidants demonstrated improvements in antioxidant markers [14]. Evidence from other studies also suggests an inverse association between high fruit consumption, especially of citrus fruits, and the risk of developing endometriosis [15]. Likewise, dietary patterns high in fruits and vegetables, as exemplified by the Alternative Healthy Eating Index (AHEI-2010), have been associated with a 13% reduction in endometriosis risk. In contrast, adherence to a Western diet, rich in processed meats, refined carbohydrates, and sweets, has been linked to a 27% increase in endometriosis risk, particularly in women without infertility [16].

Two recent systematic reviews have investigated the role of fruit consumption in relation to endometriosis. Although no statistically significant association was found between total fruit and vegetable intake and the risk of endometriosis, a general trend toward a reduced risk was observed with higher fruit consumption [17,18]. While these prior reviews provided valuable insights, one lacked statistical analysis, and the other had a narrower scope, leading to limited analytical depth regarding the relationship between fruit consumption and endometriosis [17,18]. These nuanced findings underscore the necessity for further investigation into specific dietary patterns and their potential effects on endometriosis. This systematic review and meta-analysis aims to consolidate the existing evidence regarding the relationship between fruit consumption and the incidence of endometriosis, examining whether dietary modifications could provide novel preventive strategies for the condition and enhance reproductive health.

## 2. Materials and Methods

In accordance with the PRISMA (Preferred Reporting Items for Systematic Reviews and Meta-Analyses) guidelines [19], our methodology included essential procedures to maintain transparency and methodological rigor throughout the research process (Supplementary Materials S1). Also, the Meta-analysis Of Observational Studies in Epidemiology (MOOSE) guidelines [20] were followed for conducting the meta-analysis. Additionally, the protocol for this review was registered with the International Prospective Register of Systematic Reviews (PROSPERO), under the registration number CRD42024613429. This review also followed the Cochrane Handbook for Systematic Reviews [21] to ensure methodological quality and consistency throughout the study.

### 2.1. Research Question

This study aimed to assess the potential association between fruit consumption and the incidence of endometriosis.

### 2.2. Search Strategy

On 5 October 2024, a comprehensive systematic search was conducted across three prominent databases—PubMed, Embase, and Web of Science—to identify relevant studies published between 1 January 1990 and 30 September 2024. No language restrictions were applied. In alignment with the objectives of this review, we included descriptive and observational studies from global settings, as well as non-observational studies that provided data pertinent to the primary outcome. The search strategy utilized a range of keywords related to the following terms: (1) fruit; (2) endometriosis. A detailed account of the search terms and filters applied in each database is presented in Supplementary Materials S2. Additionally, analysis of the reference lists of the included studies, as well as a manual search of the Google Scholar database, were undertaken to identify additional relevant articles. The search was conducted by two independent reviewers (PR and SK) to ensure thoroughness and minimize bias. 

### 2.3. Inclusion and Exclusion Criteria

All peer-reviewed studies were included if they investigated the impact of fruit consumption on the incidence of endometriosis. Our research question was formulated by following the Patient, Exposure, Comparison, and Outcome (PECO) framework [22] as follows:

Patient: women with and without endometriosis.
  Exposure: fruit consumption (daily and weekly quantities).
  Comparator: women without endometriosis.
  Outcome: incidence of endometriosis.

The following exclusion criteria were applied: (1) studies that did not assess the primary outcome or lacked essential data; (2) animal-based research; (3) review articles, case reports, case series, brief communications, meeting abstracts, book chapters, letters, editorials, commentaries, correspondence, and study protocols.

### 2.4. Study Selection

Two independent reviewers (PR, SK) conducted an initial screening of the identified studies based on their titles and abstracts. Full-text reviews were subsequently carried out by both reviewers (PR and SK) to confirm the inclusion of relevant data. Any disagreements in the selection process were resolved through discussion or by consulting a third reviewer (FN).

### 2.5. Data Collection

Data extraction was conducted independently by two reviewers (PR, SK). Any discrepancies or disagreements were resolved through discussion with a third reviewer (FN) and by cross-checking the extracted data. The data from each included study were systematically organized into three main categories: general information (first author, publication year, country of origin, study design, and participants’ age), endometriosis-related data (number of women with and without endometriosis and diagnostic methods used), and fruit consumption data (quantities and frequency). Additionally, the studies were categorized based on distinct levels of fruit consumption and labeled as ‘a’, ‘b’, etc., to facilitate the analysis.

### 2.6. Quality Assessment

Two researchers (PR, FN) independently evaluated the methodological quality of each included study using the Joanna Briggs Institute (JBI) Clinical Appraisal Checklists for case–control, cohort, and cross-sectional studies (Supplementary Materials S3) [23]. The checklists for case–control and cohort studies comprised 10 and 11 items, respectively, designed to assess the methodological quality of the studies, while the cross-sectional checklist contained 8 items. In all three checklists, each item was rated as “Yes”, “No”, “Unclear”, or “Not applicable.” The studies that met at least 70% of the criteria (i.e., answered “Yes” to 70% or more of the items) were considered to have a low risk of bias.

### 2.7. Quality of Evidence Assessment

The quality of evidence was assessed using the GRADE (Grading of Recommendations, Assessment, Development, and Evaluations) tool. This tool enabled the assessment of the overall quality of the evidence across studies and helped determine the confidence level in the effect estimates presented.

### 2.8. Meta-Analysis

The meta-analysis was conducted using data extracted from the eligible studies, which were initially compiled into a Microsoft Excel spreadsheet and subsequently transferred to the STATA MP 18 statistical software (Stata Corp. LLC, College Station, TX, USA). The meta-analysis was conducted for two distinct purposes. Firstly, it explored the daily fruit consumption in relation to the number of women diagnosed with endometriosis versus those without the condition. This approach allowed for the investigation of whether the frequency of daily fruit intake influenced the risk or prevalence of endometriosis. Secondly, the analysis examined the weekly fruit consumption patterns, comparing women with and without endometriosis across different ranges of weekly intake. The goal was to identify any associations between weekly fruit consumption and endometriosis risk. The defined categories of fruit consumption levels were applied in the meta-analysis to systematically compare and pool the results across studies. For each category, the effect size was computed and expressed as an unadjusted odds ratio (OR) with the corresponding 95% confidence interval (CI). A random-effects model using the restricted maximum likelihood (REML) method was employed for pooling the results across studies. The choice of a random-effects model was informed by the anticipated heterogeneity across studies due to varying populations and exposure levels.

Heterogeneity was quantified using the Cochrane Q statistic, and its magnitude was assessed using the I^2^ statistic. An I^2^ value exceeding 50% was considered indicative of substantial heterogeneity. Graphical representations of the study results were obtained using forest plots, while heterogeneity was further explored using Galbraith plots. Sensitivity analyses were conducted via a leave-one-out approach to determine the robustness of the results and the impact of individual studies on the pooled estimates.

Publication bias was evaluated using both qualitative and quantitative methods. A contour-enhanced funnel plot was generated to visually assess asymmetry, while Begg’s nonparametric rank correlation test [24] and Egger’s regression test [25] were used to statistically evaluate the presence of small-study effects. A *p*-value less than 0.05 in these tests was interpreted as indicative of potential publication bias. Where such bias was detected, Duval and Tweedie’s trim-and-fill method was planned to be applied to adjust the pooled estimates accordingly [26].

## 3. Results

### 3.1. Search Results

**Figure 1:**
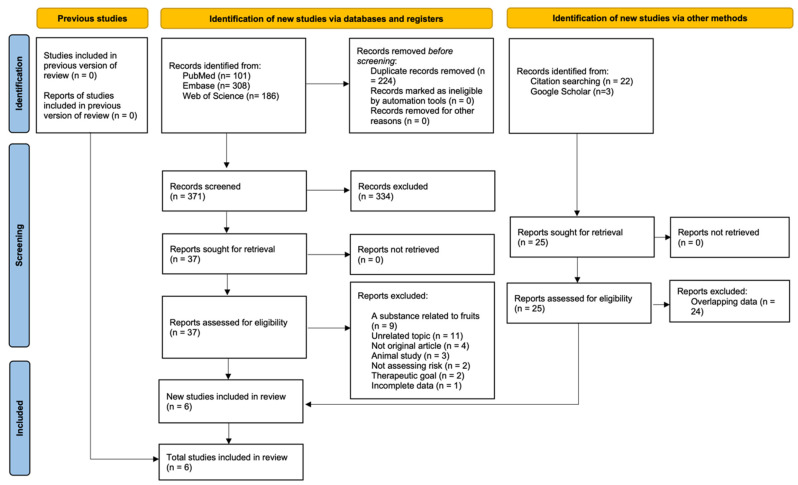
PRISMA flow diagram.

A systematic search of electronic databases yielded 595 records. After removing 224 duplicate entries, 371 unique records remained. Following a review of the titles and abstracts, 334 records were excluded, resulting in 37 articles that underwent comprehensive full-text evaluation. Ultimately, five articles satisfied the inclusion criteria and were incorporated into the meta-analysis [15,27,28,29,30]. Furthermore, a manual review of the reference lists of the included studies and an additional search in the Google Scholar database identified one more eligible article [31], leading to a total of six studies included in this systematic review [15,27,28,29,30,31]. The screening process is illustrated in the PRISMA 2020 flow diagram shown in Figure 1.

### 3.2. Study Characteristics

The six studies included in this review comprised a total of 3689 women with endometriosis and 1463 women without the condition. The sample sizes across these studies varied significantly, ranging from 19 to 2609 participants. Five of the studies included a comparison group [27,28,29,30,31], while one did not [15]. Despite the absence of a comparison group, the mentioned study reported hazard ratios and 95% confidence intervals for laparoscopically confirmed endometriosis based on daily fruit intake. Therefore, this study was included in our analysis due to its relevant data [15]. The predominant study design was case–control, with publication dates spanning from 2004 to 2024. The consumption of fruits was reported in either daily or weekly units. A comprehensive overview of the characteristics of the included studies is presented in Table 1.

### 3.3. Review of the Findings

The association between fruit consumption and the risk of endometriosis has been explored in several studies, yielding diverse findings [15,27,28,29,30,31]. A case–control study conducted in Iran identified a significant inverse relationship between fresh fruit intake and endometriosis risk, with a crude OR of 0.71 (95% CI: 0.54–0.94) [27]. Similarly, the Nurses’ Health Study II, a large prospective cohort study involving 2609 premenopausal women, demonstrated a non-linear inverse association between fruit consumption and laparoscopically confirmed endometriosis, particularly for the consumption of citrus fruits. Women consuming at least one serving of citrus fruits per day exhibited a 22% lower risk compared to those consuming less than one serving per week (95% CI: 0.69–0.89; Ptrend = 0.004) [15]. Additionally, Italian case–control studies reported a protective effect of fresh fruit intake, with an OR of 0.6 for the highest tertile of consumption compared to the lowest [29].

Contradictory findings were reported in other investigations. A Bangladeshi case–control study found no significant association between fruit consumption and endometriosis risk (*p* > 0.05) [28]. Similarly, a study assessing dietary patterns among newly diagnosed endometriosis patients and healthy controls revealed no specific link between fruit intake and endometriosis risk, although it highlighted broader unbalanced dietary habits in the affected individuals [31]. Conversely, a U.S.-based population case–control study yielded unexpected results, associating increased fruit consumption with a higher risk of endometriosis. For instance, consuming 1–2 servings of fruit daily compared to ≤1 serving was associated with an OR of 1.1 (95% CI: 0.8–1.6), and consuming more than two servings daily compared to ≤1 serving was linked to an OR of 1.5 (95% CI: 1.0–2.3, P-trend = 0.04) [30].

Overall, the evidence suggests a complex and multifaceted relationship between fruit consumption and endometriosis risk. While certain studies support a protective role of fresh or citrus fruits, others report null or adverse associations, emphasizing the need for further research to clarify these inconsistencies. Differences in study design, population characteristics, and dietary assessment methodologies likely contribute to the observed variations [15,27,28,29,30,31].

### 3.4. Quality Assessment

Our assessment indicated that four studies met a minimum of 70% of the criteria specified in the JBI Clinical Appraisal Checklists [15,27,29,30]. The remaining two studies were categorized at moderate risk of bias [28,31] (Table 1). Additional information concerning the quality assessment of the reviewed studies can be found in Supplementary Materials S4.

### 3.5. Quality of Evidence Assessment

The quality of evidence assessment performed using the GRADE Profiler version 3.6 revealed very low quality for both daily and weekly categories of fruit consumption in women with and without endometriosis. This finding indicates that the evidence should be interpreted with caution, as it may not provide a reliable foundation for clinical practice, policy decisions, or further research. Without corroborating evidence from higher-quality studies, the conclusions drawn from this analysis remain uncertain and should be approached with skepticism (Table 2).

### 3.6. Meta-Analysis

#### 3.6.1. Comparison of Daily Fruit Consumption in Women with and Without Endometriosis

**Figure 2:**
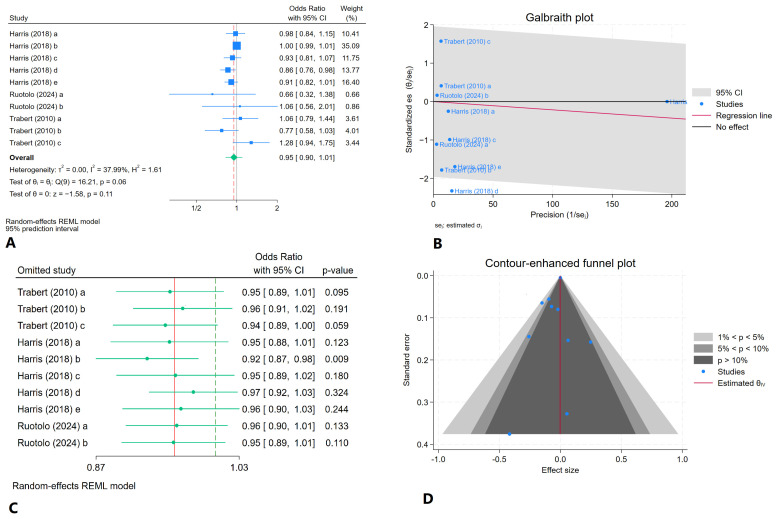
Findings of the meta-analysis of ten categories across three studies for estimating the pooled odds ratio of daily fruit consumption in women with and without endometriosis: (**A**) forest plot, (**B**) Galbraith plot, (**C**) leave-one-out sensitivity analysis, (**D**) contour-enhanced funnel plot [15,30,31]. All categories, including a, b, c, d, and e, which represent fruit consumption per week or day in women with or without endometriosis, are comprehensively explained in Table 1.

By pooling the data from ten categories across three studies [15,30,31], the random-effects model indicated relatively low heterogeneity among the analyzed studies, as evidenced by an I^2^ value of 37.99%. The estimated pooled odds ratio of daily fruit consumption in women with and without endometriosis was 0.95 (95% CI: 0.90–1.01). In the Galbraith plot, category d from the Harris study fell outside the 95% confidence interval, further demonstrating the high heterogeneity present. The leave-one-out sensitivity analysis produced odds ratios ranging from 0.92 to 0.96, all of which fell within the initially calculated 95% CI, suggesting the consistency of the findings despite the observed heterogeneity (Figure 2).

Our evaluation of publication bias revealed *p*-values of 0.62 for Egger’s test and 0.32 for Begg’s test, suggesting no significant publication bias among the studies included in our analysis. The contour-enhanced funnel plot illustrating these results is shown in Figure 2.

#### 3.6.2. Comparison of Weekly Fruit Consumption in Women with and Without Endometriosis

**Figure 3:**
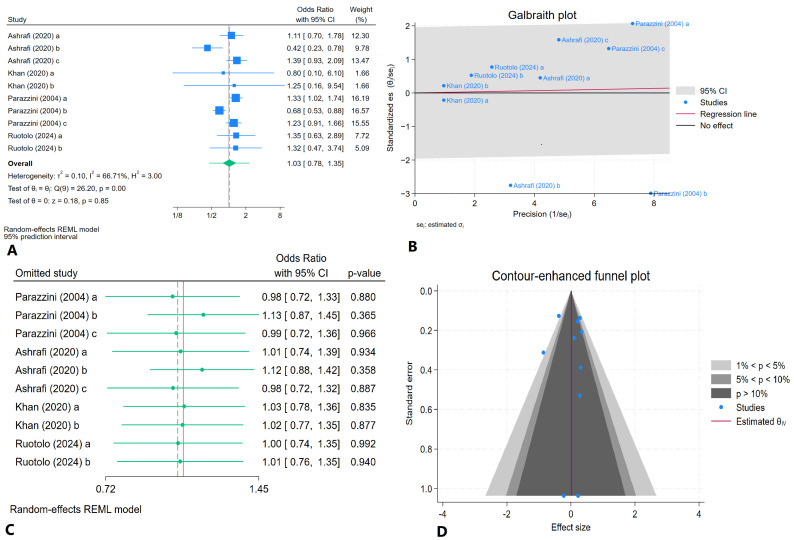
Findings of the meta-analysis of ten categories across four studies for estimating the pooled odds ratio of weekly fruit consumption in women with and without endometriosis: (**A**) forest plot, (**B**) Galbraith plot, (**C**) leave-one-out sensitivity analysis, (**D**) contour-enhanced funnel plot [27,28,29,31]. All categories, including a, b and c, which represent fruit consumption per week or day in women with or without endometriosis, are comprehensively explained in Table 1.

After pooling the data from ten categories across four studies [27,28,29,31], the analysis indicated an I^2^ value of 66.71%, signifying substantial heterogeneity among the studies. Using a random-effects model, the estimated odds ratio was 1.03 (95% CI: 0.78–1.35). Furthermore, two of the ten categories fell outside the 95% confidence interval range in the Galbraith plot. The leave-one-out sensitivity analysis yielded consistent findings, with odds ratios for weekly fruit consumption in women with and without endometriosis ranging from 0.98 to 1.13 (Figure 3).

In assessing publication bias, we obtained *p*-values of 0.103 and 0.160 for Egger’s and Begg’s tests, respectively, indicating no significant publication bias among the studies analyzed. The contour-enhanced funnel plot illustrating this assessment is shown in Figure 3.

## 4. Discussion

The present study sought to assess the association between fruit consumption and endometriosis risk by analyzing daily and weekly intake patterns across various populations. Our results showed no significant link between daily fruit intake and endometriosis risk, with a pooled OR of 0.95 (95% CI: 0.90–1.01) and moderate heterogeneity (I^2^ = 37.99%). Weekly fruit intake also showed no clear association, with an OR of 1.03 (95% CI: 0.78–1.35) and higher heterogeneity (I^2^ = 66.71%). Although prior research suggested potential connections between diet and endometriosis [32], our findings indicate that the general fruit intake may not exert a major impact on endometriosis development.

Among the six studies included in this meta-analysis, that of Trabert et al. reported a positive association between fruit consumption and an increased risk of endometriosis [30]. This finding was hypothesized to result from higher exposure to pesticides through fruit consumption. Evidence from laboratory analyses indicates that pesticide residues can persist after both harvesting and processing, with domestic fruits showing higher levels of pesticide contamination compared to imported fruits and vegetables [33,34]. Experimental studies have also demonstrated that certain pesticides exhibit estrogenic properties in both in vitro and in vivo settings [35,36]. Furthermore, animal studies involving rats with induced endometriosis revealed that exposure to high concentrations of pesticides commonly applied to orchard fruits exacerbated endometriotic lesions and contributed to recurrence [37]. Trabert et al. also identified a correlation between β-carotene intake and elevated endometriosis risk, which may reflect the higher fruit consumption observed in their study [30]. Additionally, fruits, particularly those with high glycemic indices, contain natural sugars such as sucrose, fructose, and glucose, which can cause spikes in blood sugar and insulin levels; overconsumption of these simple sugars is a significant contributor to obesity and related diseases [38,39,40,41]. The chronic elevation of insulin may contribute to systemic inflammation, potentially exacerbating the inflammatory processes associated with endometriosis [42,43]. Moreover, the study by Khan et al. found no significant relationship between fruit consumption and endometriosis risk, a result the authors attributed to the study’s single-center design and limited sample size [28]. The study by Ruotolo, while demonstrating no significant relationship between fruit consumption and endometriosis risk, concluded that fruit intake may contribute to balancing pro-inflammatory and anti-inflammatory substances, potentially reducing inflammation associated with the disease [31]. However, the remaining three studies in our analysis suggested that higher fruit consumption may reduce endometriosis risk [15,27,29].

Beyond general consumption, studies have examined specific fruit-derived compounds with potential anti-inflammatory and antioxidative effects on endometriosis. Fisetin, a polyphenol, may reduce endometriosis by modulating the nucleotide-binding domain, leucine-rich-repeat-containing family, pyrin domain-containing-3 (NLRP-3) inflammasome pathway and reducing oxidative stress in mast cells [44]. Resveratrol, commonly found in grapes, showed potential for reducing the expression of vascular endothelial growth factor (VEGF) and tumor necrosis factor alpha (TNF-α), which are key factors in inflammation and angiogenesis [45]. Maqian essential oil inhibited ectopic endometrial stromal cells, possibly due to its anti-inflammatory properties [46], while alpinumisoflavone disrupted calcium balance and mitochondrial function to suppress endometrial cell survival [47]. Apigenin and luteolin, two flavonoids, promoted apoptosis in endometriotic cells and inhibited pro-inflammatory macrophage activity, indicating additional anti-inflammatory benefits [48,49]. Fruits, which are also rich in pro-vitamin A compounds like alpha-carotene, beta-carotene, and beta-cryptoxanthin, have been found to be beneficial, as studies report that women with endometriosis tend to consume lower levels of vitamin A compared to those without the condition [14]. Dietary studies in Iranian women found that a higher phytoestrogen and flavonoid intake was associated with a lower risk of endometriosis, likely due to estrogen-modulating effects [50,51]. Moreover, sea buckthorn (*Hippophae rhamnoides* L.), abundant in flavonoids such as isorhamnetin and quercetin, carotenoids, and omega fatty acids, has shown promise in managing gynecological conditions, including endometriosis, with its berries historically used in both culinary and medicinal practices [52]. Finally, an animal study demonstrated that vitamin C, commonly found in many fruits, is effective in preventing and regressing endometriotic implants [53].

Several studies also highlight the therapeutic potential of fruit-derived compounds on endometriosis. For instance, red fruit (Pandanus conoideus) inhibited lesion development by downregulating nuclear factor kappa B (NF-kB) and VEGF expression, suggesting anti-inflammatory and anti-angiogenic effects [54]. Delphinidin, found in berries, induced apoptosis in endometrial cells by altering calcium levels and mitochondrial membrane potential [55]. Additionally, Maqian essential oil modified protein expression in ectopic endometrial cells, affecting inflammation and cell cycle regulation [56]. These findings suggest that fruit compounds may offer promising mechanisms for endometriosis management. Moreover, Schwartz et al. reported that diets high in fruit fiber were linked to a lower endometriosis risk, whereas high glycemic loads correlated with an increased risk, potentially due to insulin resistance and inflammatory pathways [57]. Collectively, these findings highlight the complex interaction between diet, inflammation, and hormonal regulation in endometriosis and suggest that specific fruit compounds may provide valuable adjunctive therapies for managing this condition.

Our meta-analysis indicates that the general fruit intake may not significantly influence endometriosis risk. While prior research suggested potential associations between dietary patterns and endometriosis, our findings align with those of Arab et al. (2022), who conducted a systematic review of four studies and reported no significant association between total fruit intake and endometriosis risk, with a relative risk of 0.97 (95% CI: 0.92 to 1.02; *p* = 0.209) [17]. Furthermore, Arab et al. noted substantial heterogeneity among the analyzed studies, with an I^2^ statistic of 85.1% (*p* < 0.001), reflecting considerable variability in outcomes across different populations and methodologies [17]. Our assessment of the quality of evidence, using the GRADE tool, classified the overall evidence as very low quality, highlighting potential limitations in the reliability of the current findings. This low evidence quality emphasizes the need for future research to focus on robust study designs, larger sample sizes, and standardized assessment methods to strengthen the validity of dietary recommendations for women at risk of endometriosis.

The findings from recent studies highlight important clinical implications for managing endometriosis through dietary interventions, despite our own meta-analysis revealing no significant association between fruit consumption and endometriosis risk. Many women with endometriosis often modify their diets in response to their symptoms, indicating a strong desire for dietary guidance to alleviate their condition. A pilot study indicated that these women typically exhibit low dietary quality and nutritional knowledge, suggesting that clinicians should emphasize nutritional counseling to empower patients in making informed dietary choices, even when the protective role of specific foods like fruits is not strongly supported by evidence [58]. Additionally, a cross-sectional study demonstrated that women with endometriosis frequently alter their dietary habits and daily activities to manage their symptoms, highlighting the need for personalized dietary recommendations [59]. Furthermore, while our findings showed no significant link between fruit intake and endometriosis, a Mendelian randomization analysis indicates that other dietary factors may still influence the risk of developing endometriosis. This reinforces the necessity for healthcare providers to promote a balanced diet rich in anti-inflammatory foods, as dietary patterns can still play a critical role in symptom management [60]. By integrating these insights into clinical practice, healthcare professionals can adopt a more holistic approach to endometriosis management, ultimately improving patient outcomes and quality of life, even in the absence of strong evidence linking specific food groups to risk reduction.

The present study possesses several strengths. The primary strength of this systematic review and meta-analysis lies in its broad and inclusive search strategy, which was designed to be as comprehensive as possible to maximize sensitivity and ensure the inclusion of relevant studies. Additionally, both the study selection and the quality assessment of the included studies were independently performed by two authors, thereby reducing potential reviewer bias. While prior systematic reviews have explored the potential associations between dietary patterns and endometriosis [17,18], our study offers a more comprehensive analysis. One of the earlier systematic reviews did not perform a statistical analysis, limiting its ability to investigate associations quantitatively [18]. In comparison, another review that included a meta-analysis lacked the scope and recency of our literature search [17]. Our study incorporated additional studies, facilitated by a more thorough and up-to-date search, resulting in a more robust analysis of the relationship between fruit consumption and endometriosis. Notably, our analysis distinguished between daily and weekly fruit consumption, providing more detailed and accurate insights. This methodological distinction, coupled with the lower heterogeneity observed in our results, enhances the reliability of our findings. Moreover, adherence to the PRISMA guidelines and the use of the GRADE tool further strengthen the comprehensiveness and rigor of our study. However, several limitations must be acknowledged. First, the relatively small number of included studies and the variability in sample sizes might contribute to the observed heterogeneity in results. The GRADE assessment categorized the quality of evidence for both daily and weekly fruit consumption as very low, indicating that the findings should be interpreted with caution. This low quality limits the generalizability of our conclusions to clinical practice or policy decisions. Moreover, the lack of uniformity in defining and measuring fruit intake across studies, along with inconsistent diagnostic criteria for endometriosis, may have hindered our ability to identify significant associations. Furthermore, the use of unadjusted values for ORs in the included studies introduces another limitation. Without adjusting for potential confounding variables, the reported associations may be biased or incomplete, potentially affecting the robustness of the results. Consequently, without corroborative evidence from higher-quality studies, including those with adjusted analyses, the conclusions drawn from this analysis remain uncertain and warrant careful consideration.

## 5. Conclusions

In conclusion, this systematic review and meta-analysis provides evidence that the general fruit consumption does not have a significant impact on the risk of developing endometriosis. Despite previous studies suggesting potential dietary connections, our findings, supported by low-quality evidence, emphasize the need for caution in drawing definitive conclusions regarding the role of fruit intake. While specific fruit-derived compounds exhibit promising therapeutic properties that may benefit endometriosis management, the variability in study designs and dietary assessments highlights the complexity of this relationship. As such, healthcare providers should continue to focus on comprehensive dietary counseling, empowering women with endometriosis to make informed dietary choices that may alleviate symptoms. Future research efforts should aim to clarify these associations through robust methodologies and larger sample sizes, ultimately enhancing our understanding of the interplay between diet and endometriosis and improving the clinical outcomes for affected individuals.

## Figures and Tables

**Table 1:** Characteristics of the included studies.

Study	Country	Design	Confirmation of Endometriosis	Mean Age ± SD (Range); y	Number of Women with Endometriosis	Number of Women Without Endometriosis	Fruit Consumption in Woman with Endometriosis	Fruit Consumption in Woman Without Endometriosis	Main Findings on Fruit Consumption and Endometriosis Risk	Study Quality (JBI Tool)
Parazzini (2004) [29]	Italy	CC	Laparoscopy	case: (20–65) control: (20–61)	503	504	a: 6 or fewer units of fruit per week: 116 w b: between 7 and 13 units of fruit per week: 169 w c: 14 or more units of fruit per week: 218 w	a: 6 or fewer units of fruit per week: 99 w b: between 7 and 13 units of fruit per week: 139 w c: 14 or more units of fruit per week: 266 w	Inverse association	LOW ROB
Trabert (2010) [30]	USA	CC	N/A	(18–49)	284	660	a: 1 or fewer units of fruit per day: 110 w b: more than 1 to 2 units of fruit per day: 85 w c: more than 2 units of fruit per day: 89 w	a: 1 or fewer units of fruit per day: 297 w b: between 1 and 2 units of fruit per day: 165 w c: more than 2 units of fruit per day: 198 w	Positive association	Moderate ROB
Harris (2018) [15]	USA	PC	Laparoscopy	N/A	2609	-	a: fewer than 1 unit of fruit per day: 516 w b: exactly 1 unit of fruit per day: 218 w c: exactly 2 units of fruit per day: 941 w d: exactly 3 units of fruit per day: 541 w e: 4 or more units of fruit per day: 393 w	-	Inverse association	Moderate ROB
Ashrafi (2020) [27]	Iran	CC	Laparoscopy	case: 31.50 ± 5.52 control: 29.35 ± 7.00	207	206	a: 6 or fewer units of fruit per week: 143 w b: between 7 and 12 units of fruit per week: 47 w c: 13 or more units of fruit per week: 17 w	a: 6 or fewer units of fruit per week: 127 w b: between 7 and 12 units of fruit per week: 43 w c: 13 or more units of fruit per week: 36 w	Inverse association	LOW ROB
Khan (2020) [28]	Bangladesh	CC	Laparoscopy and/or laparotomy and/or sonography	N/A	6	13	a: 1 or fewer units of fruit per week: 4 w b: more than 1 unit of fruit per week: 2 w	a: 1 or fewer units of fruit per week: 8 w b: more than 1 unit of fruit per week: 5 w	No association	LOW ROB
Ruotolo (2024) [31]	Italy	CS	Transvaginal sonography and/or MRI	33 ± 4.5	80	80	a: more than one unit of fruit per day: 16 w b: one unit of fruit per day: 30 w c: at least two units of fruit per week: 19 w d: less than two units of fruit per week: 9 w e: never consumes fruit: 6 w	a: more than one unit of fruit per day: 22 w b: one unit of fruit per day: 29 w c: at least two units of fruit per week: 15 w d: less than two units of fruit per week: 7 w e: never consumes fruit: 7 w	No association	LOW ROB

Abbreviations: CC: case–control, CS: cross-sectional, JBI: Joanna Briggs Institute, MRI: magnetic resonance imaging, N/A: not available, PC: prospective cohort, ROB: risk of bias, SD: standard deviation, w: women, y: years.

**Table 2:** Assessment of evidence quality using GRADE tool.

Quality Assessment							Quality
No of Studies	Design	Risk of Bias	Inconsistency	Indirectness	Imprecision	Other Considerations	
**Daily**							
10	observational studies	serious	no serious inconsistency	no serious indirectness	no serious imprecision	none	VERY LOW
**Weekly**							
10	observational studies	serious	serious	no serious indirectness	no serious imprecision	none	VERY LOW

## Data Availability

The datasets used and/or analyzed during the current study are accessible from the corresponding author on reasonable request.

## Supplementary Materials

The following supporting information can be downloaded at: https://www.mdpi.com/article/10.3390/jcm14041246/s1, Supplementary Materials S1: PRISMA checklist; Supplementary Materials S2: Utilized keywords and filters in each online dataset; Supplementary Materials S3: Joanna Briggs Institute Clinical Appraisal Checklists; Supplementary Materials S4: Tables S1–S3. Description of data: findings of Quality Assessments based on Joanna Briggs Institute Checklists. References [15,19,27,28,29,30,31] are cited in Supplementary Materials.

## Abbreviations

AHEI	Alternative Healthy Eating Index
PRISMA	Preferred Reporting Items for Systematic Reviews and Meta-Analyses
JBI	Joanna Briggs Institute
GRADE	Grading of Recommendations, Assessment, Development, and Evaluations
OR	Odds Ratio
CI	Confidence Interval
REML	Restricted Maximum Likelihood
NLRP-3	Nucleotide-binding domain, Leucine-Rich-Repeat-Containing Family, Pyrin Domain-Containing-3
VEGF	Vascular Endothelial Growth Factor
TNF-α	Tumor Necrosis Factor alpha
NF-kB	Nuclear Factor kappa B
