# Supplementary material for: Exploring the Association Between Dietary Fruit Intake and Endometriosis: A Systematic Review and Meta-Analysis
Source: J Clin Med. 2025 Feb 13;14(4):1246. doi: 10.3390/jcm14041246 (PMC11856688; doi:10.3390/jcm14041246)
Supplement: Supplementary file 1 [file jcm-14-01246-s001.zip › Supplementary Materials S2.pdf]

Search Strategy:

Pubmed (101 records):

(Endometriosis OR Endometrioses OR Endometrioma OR Endometriomas)
AND
(Fruit* OR Berr* OR (Plant AND Capsule*) OR (Legume AND Pod) OR (Legume AND Pod) OR (Plant AND Aril))
AND
1990:2024/9/30[dp]

Web of Science (186 records):

(ALL=(Endometriosis) OR ALL=(Endometrioses) OR ALL=(Endometrioma) OR ALL=(Endometriomas))
AND
(ALL=(Fruit*) OR ALL=(Berr*) OR ALL=(Plant AND Capsule*) OR ALL=(Legume AND Pod) OR ALL=(Legume AND Pod) OR ALL=(Plant AND Aril))
AND
Timespan: 1990-01-01 to 2024-09-30 (Index Date)

Embase (308 records):

(Endometriosis OR Endometrioses OR Endometrioma OR Endometriomas)
AND
(Fruit* OR Berr* OR (Plant AND Capsule*) OR (Legume AND Pod) OR (Legume AND Pod) OR (Plant AND Aril))
AND
[1990-2024]/py

Manually, articles published on Embase database in October 2024 were excluded.
