# Supplementary material for: Exploring the Association Between Dietary Fruit Intake and Endometriosis: A Systematic Review and Meta-Analysis
Source: J Clin Med. 2025 Feb 13;14(4):1246. doi: 10.3390/jcm14041246 (PMC11856688; doi:10.3390/jcm14041246)
Supplement: Supplementary file 1 [file jcm-14-01246-s001.zip › Supplementary Materials S3.pdf]

THE JOANNA BRIGGS INSTITUTE

JBI Critical Appraisal Checklist for Case Control Studies

Reviewer______________________________________Date___________________________________

Author ______________________________________Year_________Record Number____________

	Yes	No	Unclear	Not applicable
1. Were the groups comparable other than the presence of disease in cases or the absence of disease in controls?	☐	☐	☐	☐
2. Were cases and controls matched appropriately?	☐	☐	☐	☐
3. Were the same criteria used for identification of cases and controls?	☐	☐	☐	☐
4. Was exposure measured in a standard, valid and reliable way?	☐	☐	☐	☐
5. Was exposure measured in the same way for cases and controls?	☐	☐	☐	☐
6. Were confounding factors identified?	☐	☐	☐	☐
7. Were strategies to deal with confounding factors stated?	☐	☐	☐	☐
8. Were outcomes assessed in a standard, valid and reliable way for cases and controls?	☐	☐	☐	☐
9. Was the exposure period of interest long enough to be meaningful?	☐	☐	☐	☐
10. Was appropriate statistical analysis used?	☐	☐	☐	☐

Overall appraisal: Include ☐ Exclude ☐ Seek further info ☐

Comments (Including reason for exclusion)

____

____

____

© Joanna Briggs Institute 2017

JBI CRITICAL APPRAISAL CHECKLIST FOR ANALYTICAL CROSS SECTIONAL STUDIES

Reviewer______________________________________Date______________________________

Author_______________________________________Year_________ Record Number_________

	Yes	No	Unclear	Not applicable
1. Were the criteria for inclusion in the sample clearly defined?	☐	☐	☐	☐
2. Were the study subjects and the setting described in detail?	☐	☐	☐	☐
3. Was the exposure measured in a valid and reliable way?	☐	☐	☐	☐
4. Were objective, standard criteria used for measurement of the condition?	☐	☐	☐	☐
5. Were confounding factors identified?	☐	☐	☐	☐
6. Were strategies to deal with confounding factors stated?	☐	☐	☐	☐
7. Were the outcomes measured in a valid and reliable way?	☐	☐	☐	☐
8. Was appropriate statistical analysis used?	☐	☐	☐	☐

Overall appraisal: Include ☐ Exclude ☐ Seek further info ☐

Comments (Including reason for exclusion)

____

____

____

© JBI, 2020. All rights reserved. JBI grants use of these tools for research purposes only. All other enquiries should be sent to jbisynthesis@adelaide.edu.au.

THE JOANNA BRIGGS INSTITUTE

JBI Critical Appraisal Checklist for Cohort Studies

Reviewer_______________________________________Date____________________________

Author _______________________________________Year_________Record Number______

	Yes	No	Unclear	Not applicable
1. Were the two groups similar and recruited from the same population?	☐	☐	☐	☐
2. Were the exposures measured similarly to assign people to both exposed and unexposed groups?	☐	☐	☐	☐
3. Was the exposure measured in a valid and reliable way?	☐	☐	☐	☐
4. Were confounding factors identified?	☐	☐	☐	☐
5. Were strategies to deal with confounding factors stated?	☐	☐	☐	☐
6. Were the groups/participants free of the outcome at the start of the study (or at the moment of exposure)?	☐	☐	☐	☐
7. Were the outcomes measured in a valid and reliable way?	☐	☐	☐	☐
8. Was the follow up time reported and sufficient to be long enough for outcomes to occur?	☐	☐	☐	☐
9. Was follow up complete, and if not, were the reasons to loss to follow up described and explored?	☐	☐	☐	☐
10. Were strategies to address incomplete follow up utilized?	☐	☐	☐	☐
11. Was appropriate statistical analysis used?	☐	☐	☐	☐

Overall appraisal: Include ☐ Exclude ☐ Seek further info ☐

Comments (Including reason for exclusion)

____

____

____

© Joanna Briggs Institute 2017
