# Supplementary material for: Exploring the Association Between Dietary Fruit Intake and Endometriosis: A Systematic Review and Meta-Analysis
Source: J Clin Med. 2025 Feb 13;14(4):1246. doi: 10.3390/jcm14041246 (PMC11856688; doi:10.3390/jcm14041246)
Supplement: Supplementary file 1 [file jcm-14-01246-s001.zip › Supplementary Materials S4.pdf]

**Table S1.** Findings of the quality assessment of the case-control studies based on the JBI Clinical Appraisal Checklist.

| JBI Clinical Appraisal Checklist | Q1 | Q2 | Q3 | Q4 | Q5 | Q6 | Q7 | Q8 | Q9 | Q10 | Overall |
|---|---|---|---|---|---|---|---|---|---|---|---|
| **Parazzini (2004)** | Yes | Yes | Unclear | Unclear | Yes | Yes | Yes | Yes | Unclear | Yes | 7 |
| **Trabert (2010)** | Yes | Yes | Yes | Unclear | Yes | Yes | Yes | Yes | Unclear | Yes | 8 |
| **Ashrafi (2020)** | Yes | Yes | Yes | Yes | Yes | Yes | Yes | Yes | Yes | Yes | 10 |
| **Khan (2020)** | Yes | Unclear | Yes | Unclear | Yes | No | No | Yes | Unclear | Yes | 5 |

**Table S2.** Findings of the quality assessment of the cohort study based on the JBI Clinical Appraisal Checklist.

| JBI Clinical Appraisal Checklist | Q1 | Q2 | Q3 | Q4 | Q5 | Q6 | Q7 | Q8 | Q9 | Q10 | Q11 | Overall |
|---|---|---|---|---|---|---|---|---|---|---|---|---|
| **Harris (2018)** | Not Applicable | Not Applicable | Yes | Yes | Yes | Unclear | Yes | Yes | Yes | Unclear | Yes | 7 |

**Table S3.** Findings of the quality assessment of the cross-sectional study based on the JBI Clinical Appraisal Checklist.

| JBI Clinical Appraisal Checklist | Q1 | Q2 | Q3 | Q4 | Q5 | Q6 | Q7 | Q8 | Overall |
|---|---|---|---|---|---|---|---|---|---|
| **Ruotolo (2024)** | Yes | Yes | Unclear | No | No | No | Yes | Yes | 4 |
